# *In silico* and *in vivo* analysis of TIPE1 expression in diffuse large B cell lymphoma

**DOI:** 10.1515/biol-2022-0099

**Published:** 2022-08-30

**Authors:** Pei Shen, Xianjuan Shen, Guo Chen, Chunmei Zhao, Hua Cai, Xinxin Xu, Yinong Duan, Xudong Wang, Shaoqing Ju

**Affiliations:** Laboratory Medicine Center, Affiliated Hospital of Nantong University, 20 Xisi Road, Nantong 226001, Jiangsu, People’s Republic of China; Research Center of Clinical Medicine, Affiliated Hospital of Nantong University, 20 Xisi Road, Nantong 226001, Jiangsu, People’s Republic of China; Department of Pathogen Biology, School of Medicine, Nantong University, 19 Qixiu Road, Nantong 226001, Jiangsu, People’s Republic of China

**Keywords:** TIPE1, lymphoma, diffuse large B cell lymphoma

## Abstract

TIPE1 is a gene in the TNFAIP8 family involved in immune regulation and tumorigenesis. Although previous studies demonstrated that TIPE1 might play different roles in different tumors, its expression and role in lymphoma are unclear. Here we observed TIPE1 expression in diffuse large B cell lymphoma (DLBCL). Two microarrays containing 96 tumor tissue specimens were obtained from the Affiliated Hospital of Nantong University biobank. All specimens came from patients with a clear pathological diagnosis of lymphoma, lymphadenitis, breast cancer, or bladder cancer, and we performed immunohistochemical experiments on these tissue specimens. GEPIA and TIMER platforms were used for bioinformatic analyses. We found higher TIPE1 expression in tumor tissues from patients with lymphoma compared with those with lymphadenitis, breast cancer, or bladder cancer. The GEPIA and TIMER analyses revealed that TIPE1 was upregulated in DLBCL tissues but not in invasive breast carcinoma, urothelial bladder carcinoma, or liver hepatocellular carcinoma tissues. TIPE1 expression was irrelevant for pathological stage, overall survival, or DLBCL immune infiltration levels. However, TIPE1 expression was correlated with MKI67 expression in DLBCL. Overall, TIPE1’s high expression levels in DLBCL may contribute to tumor growth in DLBCL.

## 1 Introduction

Lymphoma is a malignant tumor exhibiting painless progressive lymph node enlargement and local mass lesions. It is usually classified into two categories, Hodgkin lymphoma (HL) and non-Hodgkin lymphoma (NHL), the latter of which can be further classified into B cell, T cell, and NK cell varieties according to the immunophenotype [1,2]. In NHL, diffuse large B cell lymphoma (DLBCL) is the most common lymphoma and often exhibits heterogeneous gene expression and leads to heterogeneous clinical outcomes [3,4,5,6]. In a Chinese population, polymorphism of tumor necrosis factor-alpha-induced protein (TNFAIP8, rs1045241C > T) may make the Chinese population particularly susceptible to NHL [7]. TNFAIP8 belongs to the TNFAIP8 family, which may be associated with carcinogenesis and inflammation [8,9].

TNFAIP8 gene family contain TNFAIP8, TIPE1, TIPE2, and TIPE3. The protein sequences of all four members show high homology [10]. Of these genes, TNFAIP8, TIPE1, and TIPE3 are known to greatly influence tumor occurrence and development, while TIPE2 is mainly involved in innate immunity and acquired immunity [11]. The expression of TNFAIP8 (also known as SCC-S2) has been found to be greater in invasive ductal breast carcinoma (BRCA) than in the adjacent tissues, suggesting that TNFAIP8 promotes the growth and migration of breast cancer cells [12,13]. As a phosphoinositide transfer protein, TIPE3 is highly expressed in tumor tissues of patients with lung and esophageal cancer possibly binding to phosphoinositides to promote tumor formation [11]. Contrary to what is seen with TNFAIP8 and TIPE3, TIPE2 expression is a lower expression in human non-small cell lung cancer tissues than in normal lung tissues [14]. Li et al. confirmed that over-expression of TIPE2 may inhibit cell proliferation and cell invasion of lung cancer cell lines H1299 and A549 cells [14]. TIPE2 was also downregulated in breast cancer cells, suggesting that TIPE2 can suppress the proliferation and migration of breast cancer cells [15]. This body of research shows that TNFAIP8 and TIPE3 may act as oncogenes, while TIPE2 is likely a tumor suppressor gene.

TIPE1, which may be involved in immune regulation and tumorigenesis, is known to be downregulated in ovarian cancer tissues, suggesting that TIPE1 may suppress the metastasis and tumorigenesis of ovarian cancer [16]. However, TIPE1 can be upregulated in nasopharyngeal carcinoma, suggesting a role in promoting the proliferation of nasopharyngeal carcinoma cells [17]. Hence, TIPE1 may play various important roles in different tumors. Some studies have not detected TIPE1 expression in mature lymphocytes [18]. However, TIPE1 is highly expressed in HMy2.CIR (a human B lymphoblast cell line) transformed with Epstein-Barr Viral DNA as well as in EL4 (a murine T cell line) [18]. The expression and potential role of TIPE1 in the development of lymphoma remain unclear. In this study, we observed TIPE1 expression in DLBCL, a type of B-cell lymphoma. Expression levels of TIPE1 in DLBCL and its correlation with immune infiltration level and survival, and MKI67 expression in DLBCL were analyzed using bioinformatics based on TCGA database and so on. These studies will provide basis for the further study of the role of TIPE1 in DLBCL.

## 2 Materials and methods

### 2.1 Patients

Two microarrays containing a total of 96 tumor tissue specimens from 96 patients were obtained from the biobank of the Affiliated Hospital of Nantong University. All samples came from patients who went to the hospital from March 2004 to December 2013 with a clear pathological diagnosis of HL, NHL, lymphadenitis, breast cancer, or bladder cancer.


**Informed consent:** All data were fully anonymized and no other individual information was collected from patients in this study.
**Ethical approval:** The research related to human use has been complied with all the relevant national regulations, institutional policies and in accordance with the tenets of the Helsinki Declaration, and has been approved by the Ethics Committee of Affiliated Hospital of Nantong University (Approval number: 2016056).

### 2.2 Immunohistochemical experiments

The tissue microarrays were prepared as normal paraffin-embedded sections and were dewaxed in dimethylbenzene followed by heating at 90°C in an antigen retrieval solution (Dako, Denmark). Tissue microarrays were then treated with H_2_O_2_ for 15 min at room temperature. After being inoculated with donkey serum (Solarbio, China) for blocking, a TIPE1 antibody (1:100, Santa Cruz Biotechnology, USA) was added to the samples, which were then incubated at 4°C overnight. An associated secondary antibody (Santa Cruz Biotechnology, USA) was added and incubated at 37°C for 2 h. Subsequently, DAB color development (Solarbio, China) was performed according to the instructions, and images of the tissue microarrays were captured using a Leica DM5000 B microscope (Leica, Germany). Cells stained in brown were considered positive samples [9].

### 2.3 Bioinformatic analysis

The expression of TIPE1 in tumor tissues was analyzed using the Gene Expression Profiling Interactive Analysis (GEPIA2) platform (http://gepia2.cancer-pku.cn/#index). GEPIA2 is a tool to analyze RNA sequencing expression data from The Cancer Genome Atlas (TCGA) and the Genotype-Tissue Expression (GTEx) projects [19]. Using this platform, we performed differential expression analysis and survival analysis of TIPE1 expression in tumor tissues and its control tissues.

To assess the correlation between TIPE1 expression and immune infiltration level in DLBCL, we used the Gene module of Tumor Immune Estimation Resource platform (https://cistrome.shinyapps.io/timer/, TIMER) [20,21]. We also used TIMER to examine the correlation between TIPE1 and MKI67. In addition, we obtained TIPE1 expression information from The Human Protein Atlas (HPA, http://www.proteinatlas.org) [22,23,24].

## 3 Results

**Figure 1:**
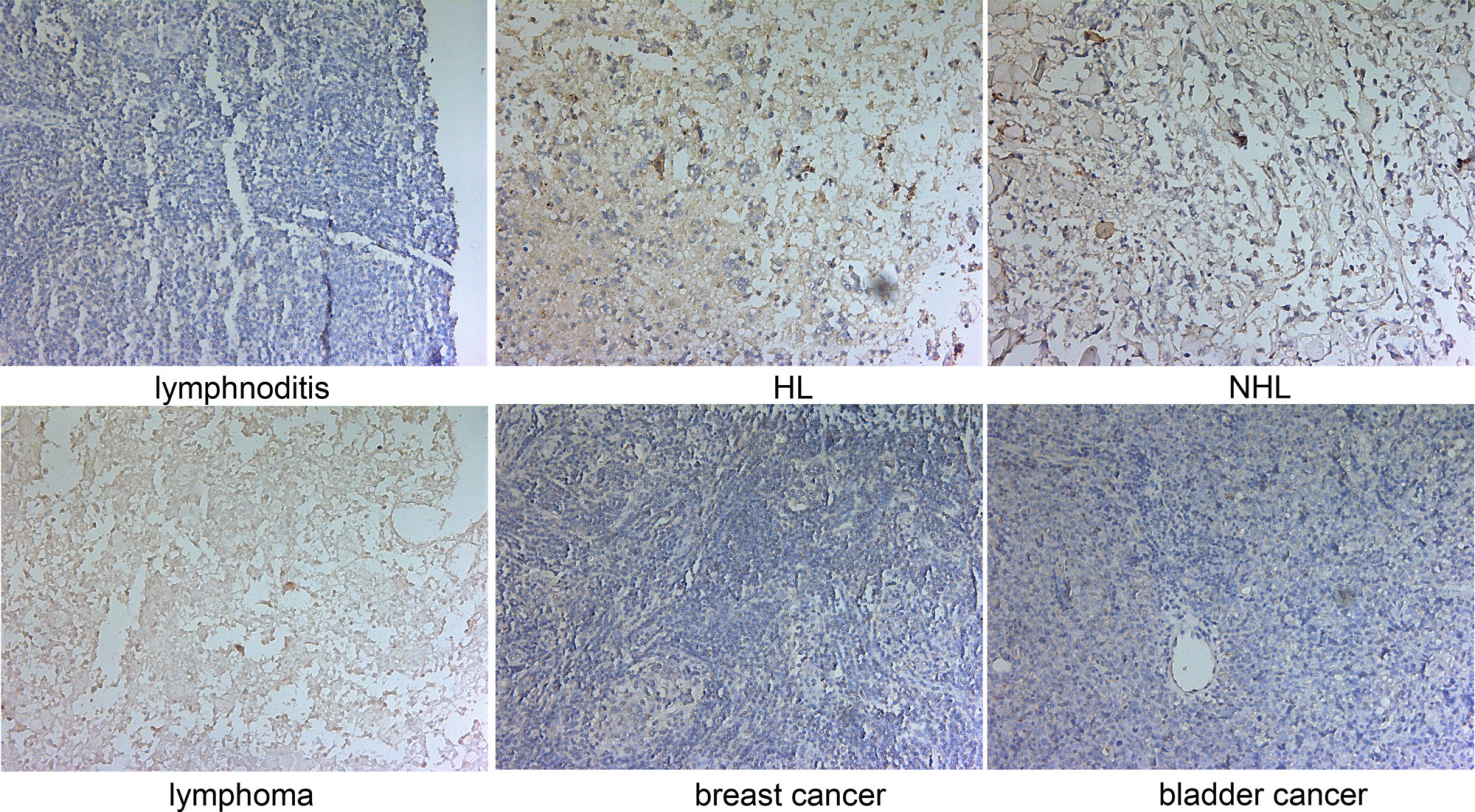
Expression of TIPE1 was upregulated in malignant lymphoma. The expression levels of TIPE1 in tissues from patients with HL and NHL were all higher than those in patients with lymphadenitis (100×). TIPE1 expression levels in tissues from patients with malignant lymphoma were higher than those in tissues from patients with lymphadenitis or other cancers (100×). Cells stained in brown were considered positive samples.

Overall, our findings suggest that TIPE1 expression is upregulated in malignant lymphomas. In the immunohistochemical experiments, the expression of TIPE1 was higher in malignant lymphomas than in lymphadenitis (Figure 1). Interestingly, TIPE1 expression from HL and NHL tissues was higher than in lymphadenitis samples. However, TIPE1 expression did not differ between HL tissues and NHL tissues. Furthermore, TIPE1 was also lower in breast and bladder cancer than in malignant lymphomas.

**Figure 2:**
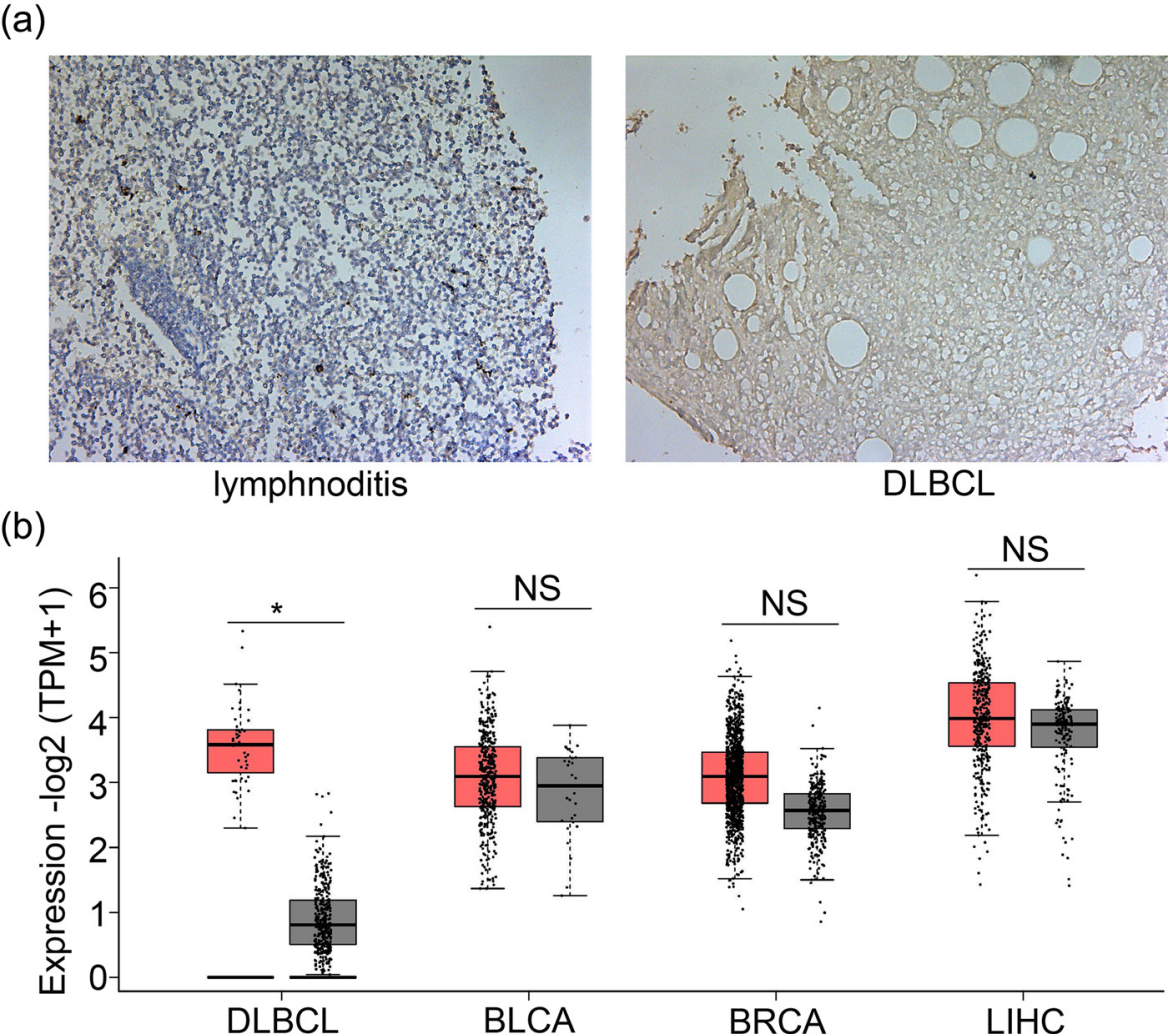
Expression of TIPE1 was upregulated in DLBCL. (a) TIPE1 expression levels in tissues from patients with DLBCL were higher than those in patients with lymphadenitis (100×). Cells stained in brown were considered positive samples. (b) The expression of TIPE1 was upregulated in the DLBCL group compared to that in the normal group. No difference in TIPE1 expression existed between the BLCA group and the normal group, between the BRCA group and the normal group, or between the LIHC group and the normal group. All the data were obtained from GEPIA2. Red indicates tumor groups, while gray indicates the normal group. * represents statistically significant differences (*p* < 0.05).

We also confirmed that TIPE1 expression was upregulated in tissues from DLBCL patients (Figure 2a). According to the data from GEPIA2, the expression of TIPE1 (Gene ID: ENSG00000185361.8) in DLBCL (*n* = 47) was higher than that in the normal group (*n* = 337, *p* < 0.05) (Figure 2b). TIPE1 expression did not differ between the invasive BRCA group (*n* = 1,085) and normal group (*n* = 291, *p* > 0.05), nor between the urothelial bladder carcinoma (BLCA) group (*n* = 404) and normal group (*n* = 28, *p* > 0.05) or liver hepatocellular carcinoma (LIHC) group (*n* = 369) and normal group (*n* = 160, *p* > 0.05).

**Figure 3:**
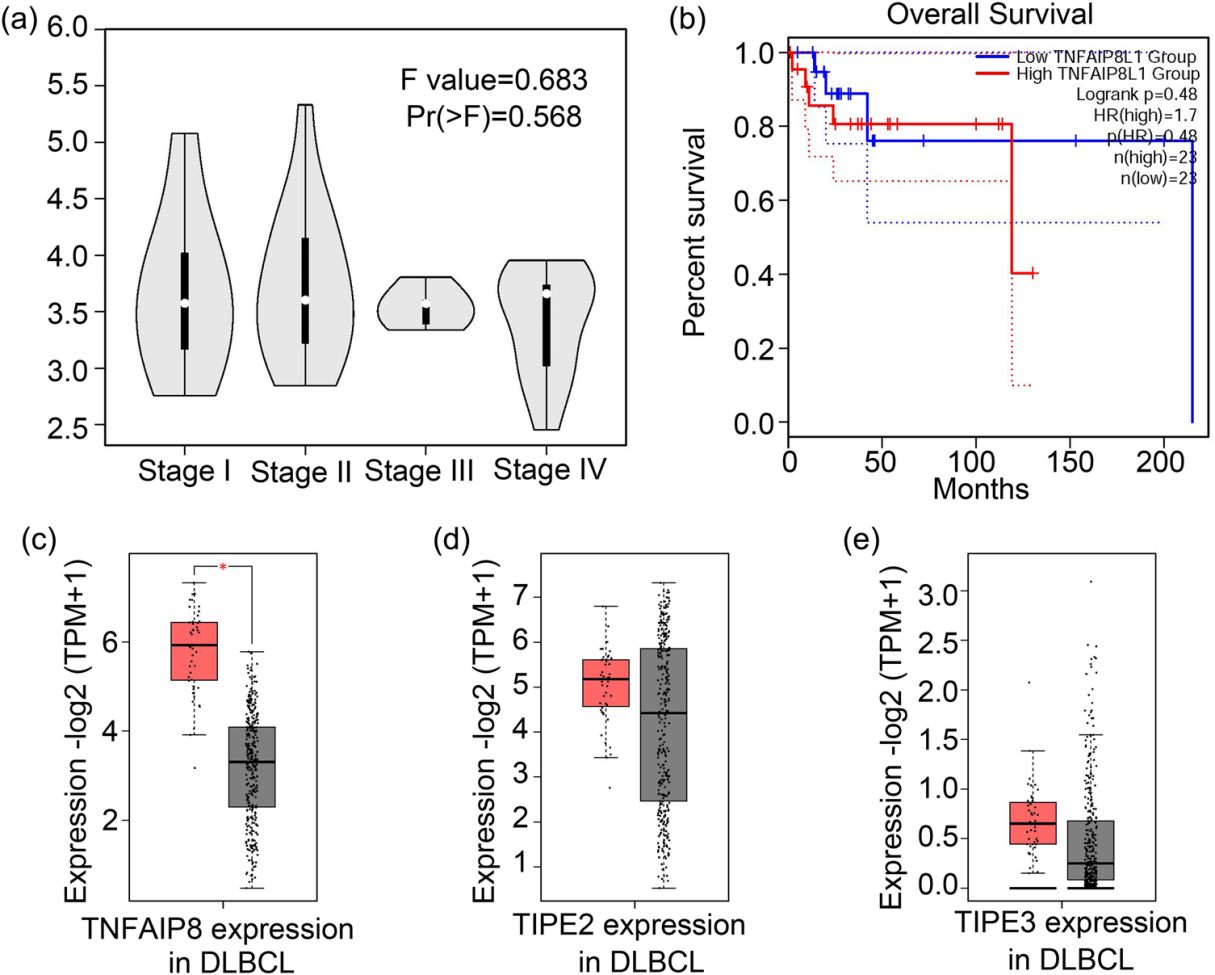
TIPE1 expression was unrelated to the pathological stage and overall survival of DLBCL. (a) TIPE1 expression and pathological stages of DLBCL were uncorrelated. (b) TIPE1 expression and overall survival were uncorrelated (*p* = 0.48). (c) The expression of TNFAIP8 was higher in DLBCL (*p* < 0.05). (d) and (e) Expression of TIPE2 and TIPE3, respectively, was not greater in DLBCL compared with those in the normal group. Red indicates tumor groups, while gray indicates the normal group.

**Figure A1:**
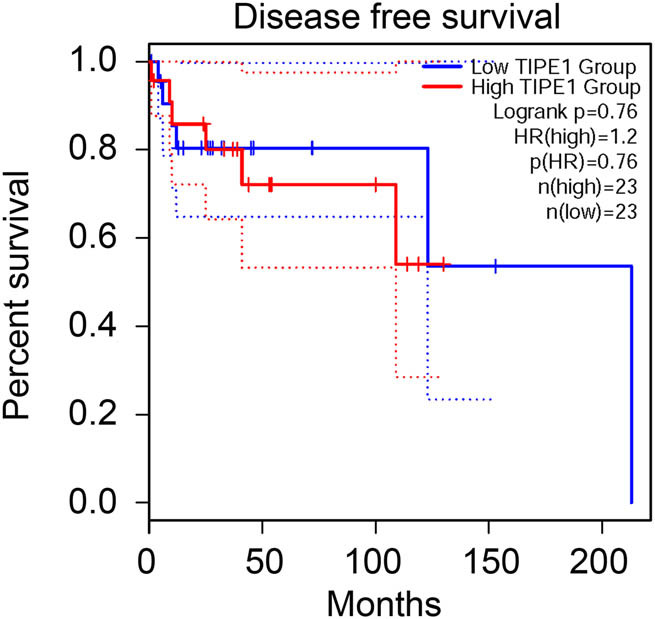
TIPE1 expression did not predict disease-free survival of DLBCL. TIPE1 expression was uncorrelated with overall survival (*p* = 0.76).

TIPE1 expression was not correlated with pathological stage nor overall survival of DLBCL. Using the GEPIA2 platform, we found that the expression of TIPE1 was independent of the DLBCL pathological stage of the tissue (Figure 3a, *F* = 0.683, Pr(*F*) = 0.568). Furthermore, overall survival was unrelated to TIPE1 expression (Log rank *p* = 0.48, *p*(HR) = 0.48, Figure 3b). Meanwhile, TIPE1 expression did not predict disease-free survival of DLBCL (Figure A1). In addition, according to the data from GEPIA2, TNFAIP8 expression was upregulated in DLBCL tumor (*n* = 47, *p* < 0.05, Figure 3c), similar to that of TIPE1 (Figure 2b). However, TIPE2 and TIPE3 expression did not differ between DLBCL tumor group (*n* = 47) and normal group (*n* = 337) (*p* > 0.05, Figure 3d and e).

**Figure 4:**
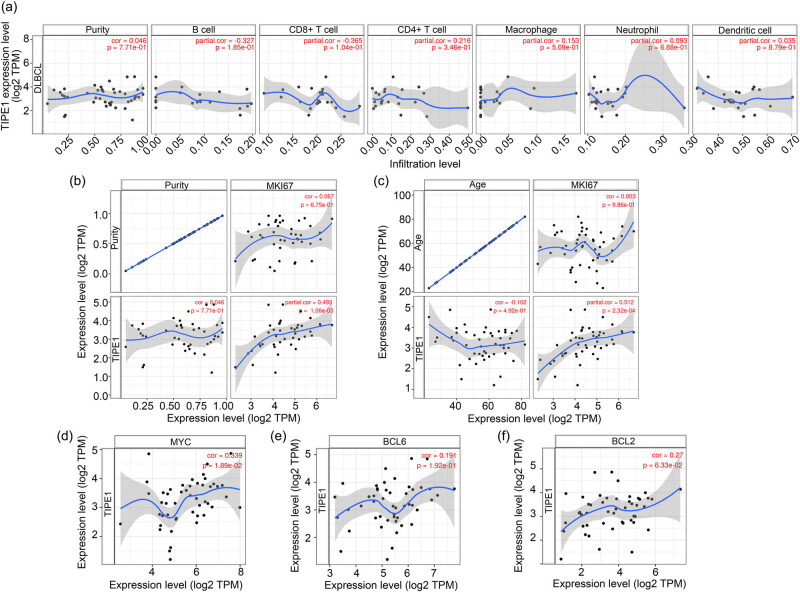
TIPE1 expression was unrelated to immune infiltration levels but correlated with MKI67 in DLBCL. (a) TIPE1 expression and immune infiltration levels of B cells, CD8 + T cells, CD4 + T cells, macrophages, neutrophils, and dendritic cells in DLBCL were uncorrelated. (b) and (c) TIPE1 expression was correlated to MKI67 expression in DLBCL (b, purity adjustment and c, age adjustment, *p* < 0.05). (d), (e), and (f) TIPE1 expression was related to MYC expression in DLBCL (*p* < 0.05), but unrelated to BCL6 or BCL2 expression (*p* > 0.05).

TIPE1 expression in DLBCL was unrelated to immune infiltration levels but correlated with MKI67. Using HPA (https://www.proteinatlas.org/ENSG00000185361-TNFAIP8L1/pathology) platform, we also found that TIPE1 was an intracellular protein whose expression level is higher in the liver than in other tissues. However, its RNA expression level has low cancer and immune cell specificity [22]. Then, according to the data from TIMER, TIPE1 expression was not related to the infiltration levels of B cells (*p* = 0.185), CD8 + T cells (*p* = 0.104), CD4 + T cells (*p* = 0.346), macrophages (*p* = 0.509), neutrophils (*p* = 0.688), and dendritic cells (*p* = 0.879) (Figure 4a). As such, TIPE1 expression may be unrelated to immune infiltration in DLBCL.

**Figure A2:**
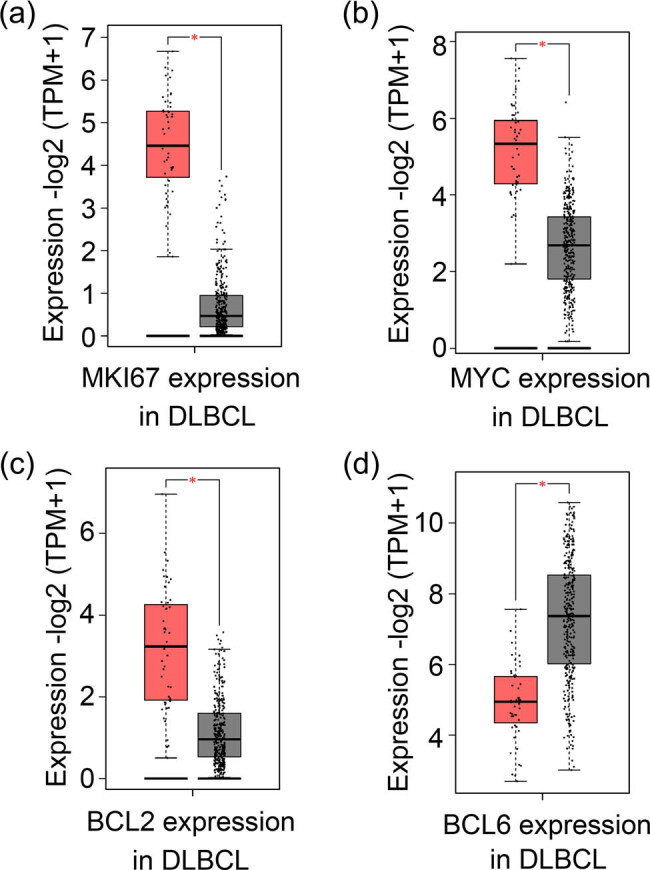
Expression of MKI67, MYC, BCL2, and BCL6 was influenced by DLBCL. MKI67 (a), MYC (b), and BCL2 (c) expression levels were upregulated in the DLBCL group compared to the normal group, while expression of BCL6 (d) was downregulated compared to the normal group. Red indicates tumor groups, while gray indicates the normal group. *represents statistically significant differences (*p* < 0.05).

Using the TIMER platform, we found that TIPE1 expression in DLBCL was predicted to correlate with MKI67 expression, whether the association was adjusted by tumor purity (*p* < 0.05, Figure 4b) or age (*p* < 0.05, Figure 4c). In addition, the expression levels of MKI67, MYC, BCL2, and BCL6 are shown in Figure A2. TIPE1 expression in DLBCL also correlated with MYC expression (*p* < 0.05, Figure 4d), but unrelated to BCL6 (*p* > 0.05, Figure 4e) or BCL2 (*p* > 0.05, Figure 4f) expression.

## 4 Discussion

According to the data from HPA in our study, we found that TIPE1 was expressed in multiple human tissues (especially in the liver) and exhibited low immune cell specificity and low human brain regional specificity. Cui et al. also reported that TIPE1 was expressed in various tissues of C57BL/6 mice, including brain neurons, liver, male and female reproductive cells, and muscle tissues as well as in tumor cells transfected with related viruses [18]. Although TIPE1 may be a prognostic marker in renal cancer, TIPE1 is typically expressed in a variety of human tumor tissues with low cancer specificity (HPA database). However, we observed that TIPE1 expression in LIHC tissues did not differ from that in normal tissues based on the data from GEPIA2. Furthermore, expression levels of TIPE1 were not upregulated in BRCA and BLCA groups (Figure 1). This differs from the report of Zhang et al., who found that TIPE1 expression was substantially reduced in hepatocellular carcinoma (HCC) tissues compared to adjacent tissues [25]. They also found that TIPE1 promoted apoptosis of HCC cells and inhibited HCC cell growth. TIPE1 may also inhibit colony formation of HCC cells and slow the growth of transplanted tumors [25]. Hu et al. found that TIPE1 inhibited the growth of breast cancer cells and demonstrated that TIPE1 expression was negatively correlated to MKI67 expression in breast cancer tissues [9]. On the other hand, TIPE1 expression was elevated, and its expression was positively correlated to MKI67 expression in cervical cancer tissues [26]. *In vitro* experiments also demonstrate that TIPE1 promotes the proliferation of cervical cancer cells through p53 pathway [26]. As such, differential expression of TIPE1 in different tumors may result in different biological functions.

In this study, all our analyses suggested that TIPE1 expression was substantially higher in the DLBCL group than in the normal group. Additionally, TNFAIP8 was found to be more highly expressed in DLBCL groups compared to the non-cancer groups, while TIPE2 and TIPE3 expression did not differ between DLBCL and normal tissues. Although TIPE1 expression was unrelated to DLBCL immune infiltration levels, DLBCL pathological stage, and overall survival of patients with DLBCL, we confirmed that MKI67 expression was upregulated in DLBCL tissues compared to that in the normal tissues based on the data from GEPIA2 (Figure A2). TIPE1 expression was also positively correlated with MKI67 in DLBCL tissues. As MKI67 is a protein associated with cell proliferation [27], these results suggest that TIPE1 expression in DLBCL may contribute to tumor growth. Previous work has shown that TIPE1/Oxi-beta can competitively bind to FBXW5 with tuberous sclerosis complex 2 (TSC2), increasing the stability of TSC2 and promoting excessive autophagy in Parkinson’s disease [28]. We also confirmed that the TIPE1 protein interacted with FBXW5 in our previous study [29]. As the autonomous autophagy of tumor cell may also promote tumor growth [30], we speculate that the higher expression of TIPE1 in DLBCL may contribute to the autophagy of tumor cells, thereby causing tumor growth of DLBCL.

In conclusion, TIPE1 is highly expressed in DLBCL and may contribute to tumor growth in DLBCL. Further study needs to be performed to observe the function of TIPE1 in DLBCL.
